# Supplementary material for: Genetically diverse uropathogenic Escherichia coli adopt a common transcriptional program in patients with UTIs
Source: eLife. 2019 Oct 21;8:e49748. doi: 10.7554/eLife.49748 (PMC6802966; doi:10.7554/eLife.49748)
Supplement: Supplementary file 1. [file elife-49748-supp1.docx]

Primers used for qPCR experiments.

| Gene | Forward primer | Reverse primer |
| --- | --- | --- |
| *cirA* | TGTTTAGGTTGAACCCTTTCGT | ACCATCGTTTCGCCATCATC |
| *fepA* | CCACCAGTGGTCAGCGT | GCTGCTTACCGGCTTGC |
| *fliA* | aacgctatgacgccctacaaggaa | agttcctgctccagttgccctatt |
| *fliC* | acagcctctcgctgatcactcaaa | gcgctgttaatacgcaagccagaa |
| *fumB* | ctgattgacgccggtaaaga | gccaagtgaacctgatggata |
| *fumC* | gctgaggaattggtgaaatc | tgacagcagagcatggttaat |
| *fyuA* | CAGACCCTGAGTGGGAAATAC | TTAACGAACCGGAAGGGAAG |
| *gapA* | CGACCTGTTAGACGCTGATTAC | CGATCAGATGACCGTCTTTCAC |
| *hma* | GGCATGACCTCAAGCAAATC | GTGCGAATAGTGAGACTGTACT |
| *sitA* | GCATGGATGTCGCCAGATAA | GCATTGCGTTGGTAGGTTTG |
| *ybtS* | CAATGGCTACCGACGATTTCT | CTGGCACCCTTTGCCTAAATA |
| *nanM* | CAGGTACGGCATGGTACAAG | TGCAGAGGTTGCTTGATCTC |
| *malK* | GGCACCTTCTTCTACCAACA | CGGCAACGAAACGCAAAT |
| *rplA* | TTTCGCCTGAGTCGGTTT | CCACACCACCATCGGTAAA |
| *rpoA* | CGGTGATGTCGAAATCGTCAAG | ACCGCGCTGAACTTTGATAC |
| *rpsA* | TTCTACGGTTGCGCCTTTAG | CTCGCAGAAGATCCGTTCAA |
